# From Mice to Men: Generation of Human Blastocyst-Like Structures *In Vitro*

**DOI:** 10.3389/fcell.2022.838356

**Published:** 2022-03-11

**Authors:** Dorian Luijkx, Vinidhra Shankar, Clemens van Blitterswijk, Stefan Giselbrecht, Erik Vrij

**Affiliations:** Department of Instructive Biomaterial Engineering, MERLN Institute for Technology-Inspired Regenerative Medicine, Maastricht University, Maastricht, Netherlands

**Keywords:** blastoids, human blastocyst-like structures, embryonic development, pluripotent stem cells, implantation

## Abstract

Advances in the field of stem cell-based models have in recent years lead to the development of blastocyst-like structures termed blastoids. Blastoids can be used to study key events in mammalian pre-implantation development, as they mimic the blastocyst morphologically and transcriptionally, can progress to the post-implantation stage and can be generated in large numbers. Blastoids were originally developed using mouse pluripotent stem cells, and since several groups have successfully generated blastocyst models of the human system. Here we provide a comparison of the mouse and human protocols with the aim of deriving the core requirements for blastoid formation, discuss the models’ current ability to mimic blastocysts and give an outlook on potential future applications.

## Introduction

In the past few years, a set of novel stem cell-based embryo models named blastoids have seen the light that recapitulate the pre-implantation blastocyst-stage embryo. Blastoids are well-poised to complement research on natural embryos as they contain both the embryonic and extraembryonic cell types, can be formed in large numbers with similar genetic makeup, are amenable to new modes of experimental manipulation including genetic screens and show features of post-implantation morphogenesis when exposed to appropriate culture conditions. In particular, human blastoids may provide promising alternatives to the use of human embryos, which are scarce and come with substantial ethical concerns (Pereira Daoud et al., 2020).

Several days after conception the blastomeric cells of the mammalian embryo compact into a tight polarized cluster named the morula. This conserved morphological event initiates, in part *via* aPKC/Hippo-mediated signaling, the first lineage bifurcation into the embryonic inner cell mass (ICM) and the extra-embryonic trophectoderm (TE), that will form the fetal placenta (Cockburn and Rossant, 2010; Gerri et al., 2020a). The TE then forms an epithelium, starts pumping fluid inside and forms a cavitated sphere encasing the ICM on one side of the cavity, which is called the blastocyst (Molè et al., 2020). Interestingly, in human, commitment to either the ICM or TE does not occur until the start of cavitation (Meistermann et al., 2021). Around embryonic day 3.5 in mouse and day 5.0 in human, the ICM undergoes a second bifurcation and specifies into the epiblast (EPI), which forms the embryo proper, and primitive endoderm [PrE; named hypoblast in human (HYPO)], which gives rise to extra-embryonic endoderm tissues including parts of the yolk sac (Molè et al., 2020). In mouse, EPI and PrE cells are initially mixed in a salt/pepper-like distribution after which the PrE segregates and forms an epithelium on top of the EPI, facing the blastocyst cavity. There is evidence of HYPO formation and subsequent segregation taking place in human embryos as well, although it is not clear whether the initial distribution of HYPO cells in the ICM follows a similar salt and pepper-like pattern as observed in mouse (Roode et al., 2012). Meanwhile the TE differentiates and can be subdivided in the polar and mural TE. The polar TE refers to the TE directly in contact with the EPI and in mouse acts as a stem cell pool for the TE, whereas the mural TE is on the opposite side of the cavity. A striking difference between mouse and human development is that the mouse blastocyst attaches to the uterine wall with the mural side, while the human blastocyst is believed to attach with the polar side (Molè et al., 2020).

Thus, phenomenologically, pre-implantation development seems largely conserved between mouse and human, however the molecular underpinnings may differ. These differences become more pronounced in early post-implantation morphogenesis when also the embryo architecture and the maternal/fetal interface deviate from mouse [reviewed in Gerri et al. (2020b), Molè et al. (2020)]. As such, there is a clear need for a sophisticated model of the human pre- and peri-implantation embryo. In addition, the wide use of mouse as a model species and subsequent vast amounts of data available on mouse embryology and gene function form a solid foundation for generating and testing embryo models (Taft, 2008). Furthermore, in contrast to human, mouse embryo models may be amenable to model full organismal development.

For faithful recapitulation of blastocysts, it is essential that blastoids contain the EPI, HYPO/PrE and TE lineages. Embryonic stem cells (ESC) (Evans and Kaufman, 1981; Martin, 1981; Thomson et al., 1998), trophoblast stem cells (TSC) (Tanaka et al., 1998; Kubaczka et al., 2014; Okae et al., 2018; Frias-Aldeguer et al., 2020), and extra-embryonic endoderm cells (XEN) (Niakan et al., 2013; Anderson et al., 2017; Linneberg-Agerholm et al., 2019) form *in vitro* analogues of the EPI, TE and PrE/HYPO, respectively, and can generally be maintained as 2D monolayer cultures. More recently, chemically defined culture conditions have been identified that permit the expansion of multiple levels of pluripotent cells that reflect different developmental states of the pre-to early post-implantation EPI (Ying et al., 2008; Theunissen et al., 2014; Yang J. et al., 2017; Yang Y. et al., 2017; Guo et al., 2017; Gao et al., 2019; Bayerl et al., 2021; Shen et al., 2021). These 2D cultures form the basis of the more complex 3D models, such as blastoids, that are designed to capture both the specification of cell types and their spatiotemporal organization. As formation of 3D models relies heavily on self-organization, the starting conditions of the 2D cultures are critical for the successful generation of 3D models. On top of the stem cells themselves and the chemical cues that direct self-organization, the 3D culture platforms play an important role in providing the freedom to re-organize, as well as enabling *in situ* imaging-based readouts and enabling high-throughput screening assays.

The first report on the generation of blastoids recapitulated the mouse blastocyst formation by using a co-culture of mouse ESCs, which resemble the pre-implantation EPI (Evans and Kaufman, 1981; Martin, 1981), and mouse TSCs, which resemble the TE (Tanaka et al., 1998). When these types were combined through sequential seeding into microwells and exposed to a mixture of growth factors and small molecule signaling pathway modulators they formed blastocyst-like structures (Rivron et al., 2018). Following this report, several other labs have reported modified methods for blastoid generation that all recapitulate the blastocysts’ overall morphology and contain the three distinct cell lineages (Rivron et al., 2018; Kime et al., 2019; Li et al., 2019; Sozen et al., 2019; Zhang et al., 2019).

Although the culture of human ESCs and later TSCs has been possible for some time (Thomson et al., 1998; Okae et al., 2018), practical and ethical restrictions have impeded advances in human embryo models. Much of the work on human models consequently relies on preceding findings in mouse. However, this gap is closing with the recent accumulation of studies in both species, including the improved understanding of the similarities and differences in pluripotency (Brons et al., 2007; Tesar et al., 2007; Weinberger et al., 2016), methods to culture blastocysts towards the post-implantation stage *in vitro* (Bedzhov et al., 2014; Deglincerti et al., 2016; Shahbazi et al., 2016), advanced single cell omics studies to demarcate cell identities and regulatory networks that underlie EPI, PrE/HYPO and TE specification (Nakamura et al., 2015; Nakamura et al., 2016; Meistermann et al., 2021), and the *in vitro* differentiation of TE and its derivatives (Castel et al., 2020; Io et al., 2021). Through the use of human extended pluripotent/expanded potential stem cells (EPSCs), induced pluripotent stem cells (iPSCs) and naïve ESCs, several groups have reported their first successes with human blastoid generation (Fan et al., 2021; Kagawa et al., 2021; Liu et al., 2021; Sozen et al., 2021; Yanagida et al., 2021; Yu et al., 2021). The methods employed have yet some marked differences between the groups, underlining the different angles and controversies surrounding the cellular subtypes and signaling pathways involved in early human development.

In this review, we aim to give an overview of the current mouse and human blastoid protocols, compare mouse and human protocols to identify core requirements for blastoid formation and discuss to what extent current blastoids mimic blastocysts.

First, we will address several of the key components in the generation of mouse and human blastoids: the starting cell lines and their flavor of pluripotency, the soluble signaling pathways modulators used to steer differentiation of cells towards the blastocyst lineages and the culture platforms applied to facilitate 3D self-organization. Additionally, we will compare the timelines of the protocols and the extent of successful lineage specification. Next, we will delineate the capacity of blastoids to recapitulate principles of blastocyst development and the potential for post-implantation progression *in vitro*. Finally, we will provide an outlook on the future applications of both mouse and human blastoids.

## Generation of Mouse and Human Blastocyst Models *In Vitro*

### Cell Sources and Flavors of Pluripotency

As delineated above, the pre-implantation mammalian blastocyst is comprised of three lineages: the TE, the PrE/HYPO and the EPI. In mouse, stem cell analogues can be derived from all these three primary cell types and propagated as self-renewing stem cells, namely TSCs (Tanaka et al., 1998), XEN cells (Kunath et al., 2005; Anderson et al., 2017), and pluripotent ESCs (Evans and Kaufman, 1981; Martin, 1981), respectively. The term pluripotency describes the capacity of cells to form all cell types of the mature body in response to developmental cues (Silva and Smith, 2008). Leukemia inhibitory factor (LIF) was originally found to maintain pluripotency in mouse ESCs (Williams et al., 1988). Since, Ying et al., identified that the addition of the two inhibitors PD0325901 (ERK/MAPK inhibitor) and CHIR99021 (GSK3β inhibitor) can sustain mouse ESCs in a so-called pluripotent ground-state, which closely reflects the late blastocyst-stage epiblast and exhibits lower cell-to-cell variability and thereby manifests in more homogenous *in vitro* cultures (Ying et al., 2008). In the mouse embryo, progression of the EPI towards the post-implantation stage is characterized by a transiently evolving transcriptional and epigenetic signature of pluripotency, from which *in vitro* analogues can be captured in discrete states (Morgani et al., 2017; Neagu et al., 2020; Kinoshita et al., 2021), including the pluripotency endpoint (i.e., primed) post-implantation EPI cells named epiblast-derived stem cells (EpiSCs) (Brons et al., 2007; Tesar et al., 2007). *In vitro* at least, the pluripotency domain appears non-linear since additional states have been identified [reviewed in Morgani et al. (2017)], including so-called extended and expanded PSCs (EPSCs) (Yang J. et al., 2017; Yang Y. et al., 2017) that, reportedly, are able to also give rise to both the extraembryonic PrE and TE cell types. Although EPSCs do robustly form PrE, transcriptomic comparisons performed by Posfai et al. contested the claim of murine EPSC’ ability to form *bona fide* TE (Posfai et al., 2021). In fact, the data suggest that EPSC do not have an additional totipotent character relative to native ESC and cluster instead closer to early post-implantation pluripotent cells (Posfai et al., 2021).

In contrast to mouse, human blastocyst-derived ESCs were originally maintained *in vitro* in a so-called primed state that reflects more the post-implantation EPI (Thomson et al., 1998) rather than the blastocyst-stage EPI. Later, numerous regulatory cocktails have been developed that claim the expansion of human ESCs and iPSCs in a naïve state (Chan et al., 2013; Gafni et al., 2013; Takashima et al., 2014; Theunissen et al., 2014; Ware et al., 2014; Bredenkamp et al., 2019b; Bayerl et al., 2021) that shares features with mouse ground-state ESCs such as their dome-like colonies, predominant activation of the OCT4 distal enhancer, germline potency and pre-X-chromosome inactivation (Weinberger et al., 2016; Devika et al., 2019; Yilmaz and Benvenisty, 2019). Moreover, methods for culturing mouse EPSCs have been translated to human as well and termed human EPSCs (Gao et al., 2019). Nevertheless, establishing a consistent and pure human naïve line can be challenging as is evidenced by current efforts to optimize the combinations of factors that permit robust expansion of human naïve pluripotent PSCs from diverse genetic backgrounds (Bredenkamp et al., 2019a; Bayerl et al., 2021; Khan et al., 2021). The progress in the field of naïve pluripotency over the past decade has been covered extensively elsewhere and will therefore not be discussed in detail here (Weinberger et al., 2015; Smith, 2017; Yilmaz and Benvenisty, 2019; Taei et al., 2020). Important to note, whereas mouse PSC cannot readily differentiate into TE lineages, human ESCs appear to have a certain capacity to do so (Xu et al., 2002; Amita et al., 2013). Although there is no consensus yet about whether primed hPSC form *bona fide* trophoblast cells or something related (e.g., mesoderm or amnion), it appears that hPSCs maintained in EPSC and naïve conditions do form stable self-renewing lines reminiscent of early post-implantation trophoblast (Castel et al., 2020; Cinkornpumin et al., 2020; Guo et al., 2021; Io et al., 2021). Robust expansion cultures of pre-implantation TE have not been reported to date, however. Besides the differentiation to trophoblast, human naïve ESCs were reported capable of differentiating into HYPO-like cells (Linneberg-Agerholm et al., 2019).

Of note, it is important that, independent of the expansion protocol that is used, the maintenance of pure and uniform pluripotent cell populations is an essential starting point to form blastoids for both mouse and human.

**FIGURE 1:**
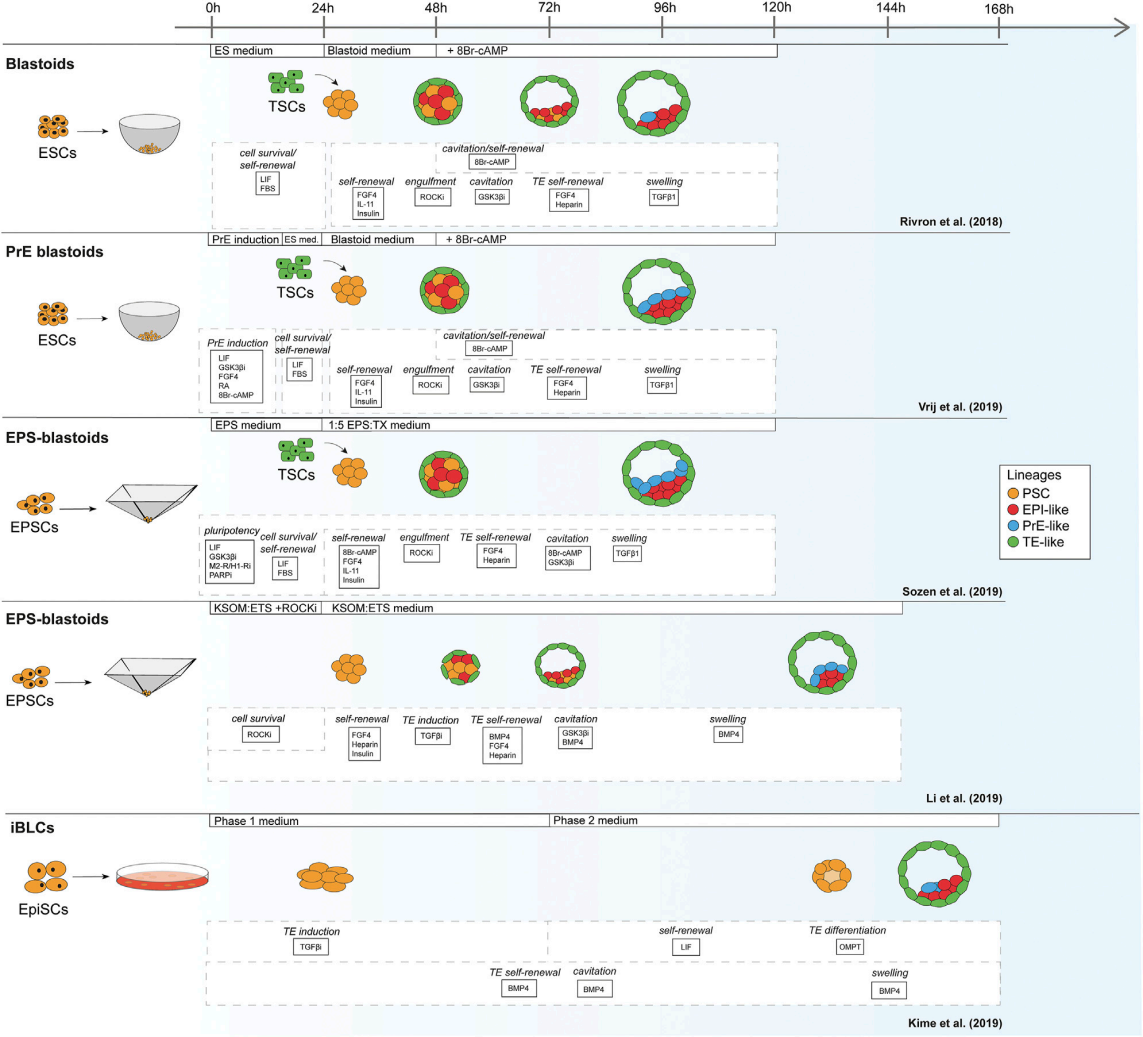
Schematic of mouse blastoid protocols. The names given are taken from the original publications.

For mouse, the first blastoids were formed using ESCs cultured in the 2i/LIF ground-state condition in order to form EPI and PrE, combined with multipotent TSCs to mimic the TE (Figure 1) (Rivron et al., 2018). In contrast to the protocols based on mouse ESCs, Li et al. reported blastoids based on murine EPSCs without addition of TSCs (EPSC-only blastoids; Table 1) (Rivron et al., 2018; Li et al., 2019; Sozen et al., 2019; Vrij et al., 2019). As mentioned before, while mouse ESCs are committed to the EPI lineage, mouse EPSCs are reportedly able to give rise to all cell types of the conceptus (Yang J. et al., 2017; Yang Y. et al., 2017). Although EPSCs do form PrE in blastocysts and in blastoids the claim of murine EPSC ability to form *bona fide* TE is contested (Posfai et al., 2021). Strikingly, although EPSC-only blastoids can form blastoids with fewer exogenous cues than the other models, the efficiency of blastoid generation appears higher when using TSCs for the TE compartment rather than TE induction from EPSCs alone (15 vs. 60% formation efficiency) (Li et al., 2019; Sozen et al., 2019). This is also supported by the findings of Kime et al. (2019). This group used their previously developed protocol for converting post-implantation mouse EPI cells to naïve mouse PSCs (Kime et al., 2016) and found that these converted cells could give rise to blastocyst-like structures on their own, albeit at a low efficiency (Kime et al., 2019).

**TABLE 1:** Overview of mouse blastoid protocols, summarizing culture conditions, experimental time lines, blastocyst marker expression, characterization experiments performed, post-implantation progression and limitations of the individual protocols.

	**Rivron, N.C., Frias-Aldeguer, J., Vrij, E.J. et al. Blastocyst-like structures generated solely from stem cells.Nature (2018)**	**Vrij, E.J., Scholte op Reimer, Y.S., Frias Aldeguer, J., et al. Chemically-defined induction of a primitive endoderm and epiblast-like niche supports post-implantation progression from blastoids. bioRxiv (2019)**	**Sozen, B., Cox, A.L., De Jonghe, J., et al. Self-Organization of Mouse Stem Cells into an Extended Potential Blastoid. Developmental Cell (2019)**	**Li, R., Zhong, C., Yu, Y., Liu, H., et al. Generation of Blastocyst-like Structures from Mouse Embryonic and Adult Cell Cultures. Cell (2019)**	**Kime, C., Kiyonari, H., Ohtsuka, S., et al. Induced 2C Expression and Implantation-Competent Blastocyst-like Cysts from Primed Pluripotent Stem Cells. Stem Cell Reports (2019)**
Cell lines	ES: V6.5, H2B-RFP V6.5 sub-clone, PDGFRα-H2B-GFP, SOX17-GFP and IB10	ES: PDGFRα-H2B-GFP/+, GATA6-H2B-Venus/+, H2B-RFP V6.5 sub-clone, ColA1 TetO-GATA4-mCherry/+, R26 M2rtTA/+TS: F4 and F1	ES: PDGFRα ESCs or EPSCs, CAG:GFP ESCs or EPSCs, ROSA-mTmG ESCs or EPSCs	EPSC: EPS1 (tdTomato+), EPS2 (tdTomato+), ES: B6N-22, B6 GFP + ES iPSC from ear fibroblasts	EpiSC: mEpiSCs, XGFP mEpiSCs with MERVL:DSRed /mCherry or EOS:DSRed/mCherry
	TS: F4, F1 and CDX2-eGFP		TS: WT- and EGFP-TSCs		
Maintenance medium	ES: 2i/Lif N2B27 medium (Ying et al., 2008)	ES: 2i/Lif N2B27 medium	ES: 2i/Lif N2B27 medium	EPSC: N2B27-LCDM (Yang et al., 2017b)	EpiSC: ND227 medium (Ying et al., 2008) + Activin A + bFGF
	TS: TX medium (Kubaczka et al., 2014)	TS: (modified) TX medium	TS: TX medium	ES: 2i/Lif N2B7 medium	
Oxygen during maintenance	20%	20%	20%	20%	20%
Platform	Agarose hydrogel microwells, 200 μm Ø	Agarose hydrogel microwells, 200 μm Ø	AggreWell™ plates 400 μm Ø	AggreWell™ plates 400 μm Ø	Fibronectin-coated 6 wells plate
Mouse blastoid protocol (Day 0 = seeding ESC/EPSC on platform)	0 h	0 h	0 h	0 h	-14-16 h
	ES medium	PrE induction medium	EPS medium	KSOM:ETS medium (1:1)	ND227 medium + Activin A + bFGF
	24 h	At 21 h ES medium	24 h	[ETS = N2B27:basal TSC medium (1:1)] + ROCKi	0 h
	Seed TSC	24 h	Seed TSC	24 h	Phase 1 medium
	Blastoid medium	Seed TSC	EPS:TX medium (1:5)	KSOM:ETS medium (1:1)	96 h
	48 h	Blastoid medium			Phase 2 medium
	Blastoid medium + 8Br-cAMP	48 h			
		Blastoid medium + 8Br-cAMP			
Oxygen during blastoid formation	20%	20%	20% during the first 24 h	20%	20%
			5% after TSC seeding		
Initial cell seeding number	5 ESCs + 12 TSCs per microwell	7 ESCs + 17 TSCs per microwell	4 E(P)SCs + 8 TSCs per microwell	5 EPSCs per microwell	30–50,000 mEpiSC cells/well
Aggregation	24 h	24 h	24 h	24 h	N/A
Cavitation	48–65 h	48–65 h	72–96 h	72 h	∼120 h
Specification of tissues	96 h: NANOG (EPI), GATA6 (PrE), CDX2 (TE)	96 h: NANOG (EPI), GATA6 (PrE)	96 h: NANOG (EPI), FOX2A (PrE), PDGFRα (PrE), CDX2 (TE)	24 h: SOX2 (ICM/EPI)	168 h: NANOG (EPI), OCT4 (EPI), GATA4/6 (PrE), PDGFRα (PrE), CDX2 (TE), TROMA-I (TE)
				48 h: active YAP (TE)	
				120–144: GATA4 (PrE)	
Formation completed	96 h	96 h	96 h	120–144 h	168 h
Yield of cavitated structures with EPI, TE and HYPO	12%	36%	15%	15% (∼2.7% for clonal EPS-blastoids)	5–30 per well
Embryonic timeline	E3.5-4.5	E3.5-4.5	E3.5-5.0	E3.5-4.5	unknown
Characterization	Immunohistochemistry qRT-PCR	Immunohistochemistry	Immunohistochemistry qRT-PCR	Immunohistochemistry sc-RNAseq	Immunohistochemistry qRT-PCR
	scRNA-seq	Post-implantation progression	scRNA-seq	bulk RNA-seq	Derivation of ES and TS
	Derivation of ES and TS cells		Post-implantation progression	Derivation of ES and TS cells	Post-implantation progression
	Injection into mouse blastocysts			Injection into mouse blastocyst	
	Post-implantation progression			Post-implantation progression	
Markers used	EPI: NANOG, OCT4, PrE: GATA6, PDGFRα	EPI: NANOG, OCT4	EPI: NANOG, OCT4	EPI: NANOG, OCT4, SOX2	EPI: NANOG, OCT4, PrE: GATA4/6, PDGFRα
	TE: CDX2, KRT18	PrE: GATA6, PDGFRα	PrE: SOX17, PDGFRα, FOX2A, GATA4, GATA6	PrE: GATA4, GATA6	TE: CDX2, GATA3, TROMA-I
			TE: CDX2, KRT8, TFAP2C	TE: CDX2, CK8, KRT8, TFAP2C	TE/ICM: YAP
Post-implantation progression (attached culture)	*In vivo* in uteri mus musculus decidualization occurred	*In vitro* on tissue-culture glass or polystyrene plastic in IVC1 medium for 96 h	*In vivo* in uteri mus musculus decidualization occurred	*in vivo* in uteri mus musculus decidualization occurred	*In vivo* in uteri mus musculus decidualization occurred in 6.7% of the cases
	CDX2, ELF5, TEAD4, HAND1, ASCL2 and proliferin (TE) positive cells observed	OCT4+ (EPI) and GATA6+ (PrE) present	PDGFRα+ (PrE), CDX2+ and KRT18+ (TE) present	*in vitro* on µ-Slide 8-well in IVC1 medium and IVC2 medium; culture for 48–96 h	TROMA-I+ (TE) cells were observed invading maternal tissue
			*In vitro* on ibidi-u plates coated with Matrigel in IVC1 medium (24 h), followed by IVC2 (next 48 h)		PL-I (visceral endoderm) present
			PDGFRα+ (PrE), GFP (TE) and OCT4+ (EPI) present		
Limitations	PrE is underdeveloped and structures arrest in post-implantation development	Structures arrest in post-implantation development	PaE is not fully formed and structures lack Reichert’s membrane	EPSCs’ ability to form TE is contested, therefore they can’t be considered equivalent to totipotent blastomeres	Low efficiency
					No high throughput platform blastoids are resorbed during post-implantation development

**FIGURE 2:**
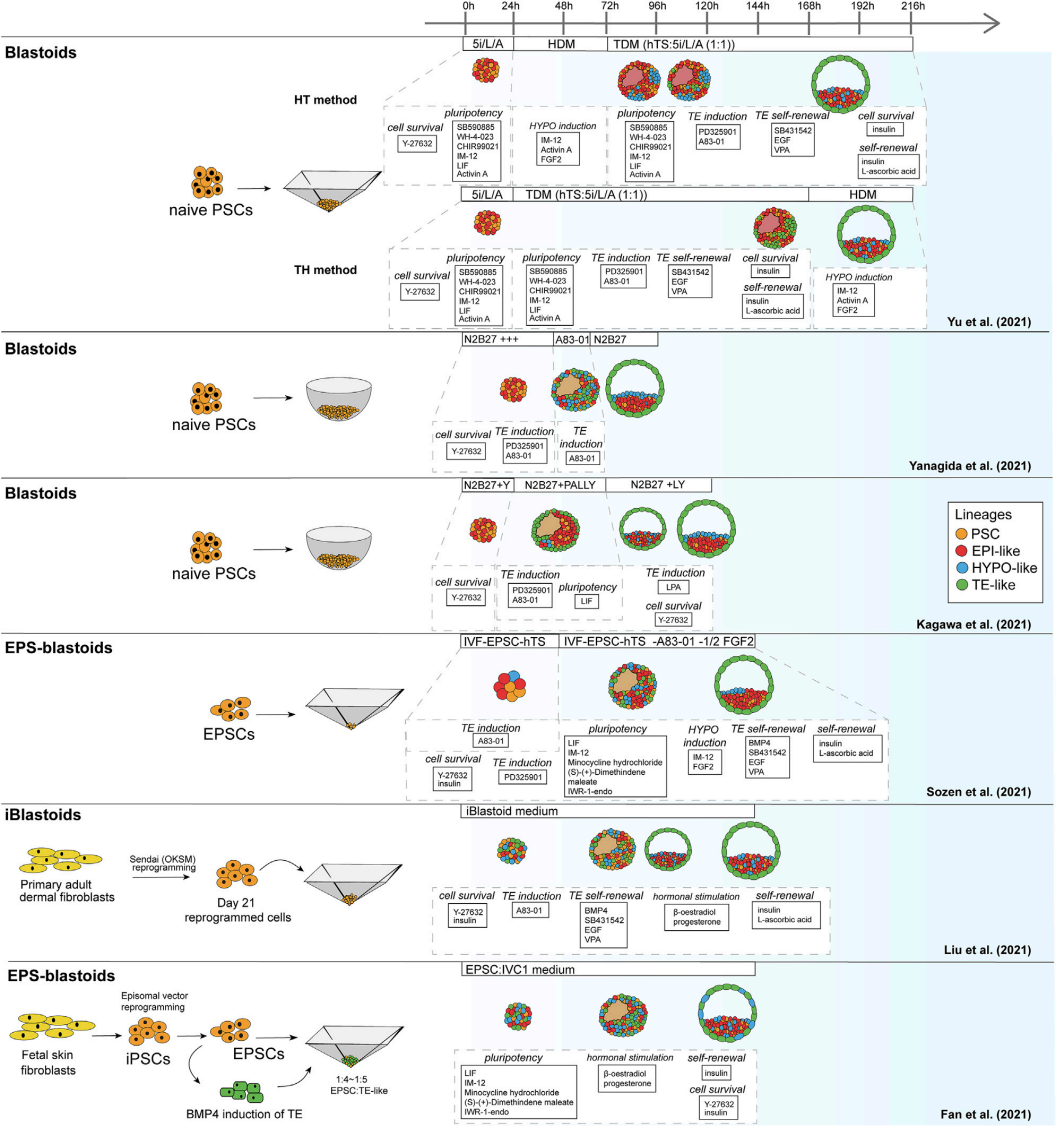
Schematic of human blastoid protocols. The names attributed to the models are taken from the original publications.

Similar to the mouse models, human blastoid models were created with various types of PSCs that are believed to confer different pluripotency states (Figure 2). While some labs use human ESCs and iPSCs grown in naïve conditions (Kagawa et al., 2021; Yanagida et al., 2021; Yu et al., 2021), others have used EPSC conditions (Fan et al., 2021; Sozen et al., 2021) and one group has used direct reprogramming of fibroblasts over a course of 21 days into cells that form blastoids (Liu et al., 2021). This diversity in starting material likely contributes to the variation between methods in blastoid generation efficiency and capacity for lineage specification (Table 2). Moreover, depending on the pluripotency state, triggers for directing the founding cells towards an organized blastocyst-like structure vary as well, as is also observed in the mouse protocols (Rivron et al., 2018; Li et al., 2019; Sozen et al., 2019; Vrij et al., 2019).

**TABLE 2:** Overview human blastoid protocols, summarizing culture conditions, experimental time lines, blastocyst marker expression.

	**Yu, L., Wei, Y. et al. Blastocyst-like structures generated from human pluripotent stem cells. Nature (2021)**	**Liu, X., Tan, J.P. et al. Modelling human blastocysts by reprogramming fibroblasts into iBlastoids. Nature (2021)**	**Yong Fan, Zhe-Ying Min, et al. Generation of human blastocyst-like structures from pluripotent stem cells. bioRxiv (2021)**	**Berna Sozen, Victoria Jorgensen. et al. Reconstructing human early embryogenesis in vitro with pluripotent stem cells. bioRxiv (2021)**		**Yanagida, A., Spindlow, D., et al. Naive stem cell blastocyst model captures human embryo lineage segregation. Cell Stem Cell (2021)**	**Kagawa, H., Javali, A., et al. Human blastoids model blastocyst development and implantation. Nature (2021**)
Cell lines	WIBR3 (OCT4-2A-GFP) hES cells, Human (fore)skin fibroblasts (neonatal origin)	Primary human adult dermal fibroblasts	Human fetal skin fibroblasts	RUES2-RLG (SOX2-GFP), H9 and ESI0017		HNES1-GATA3:mKO2, niPSC HDF75 and cR-Shef6	HNES1, H9, cR-Shef6, NIPSC16.2.b and cR-NCRM2
	**reprogrammed to iPS cells**	**reprogrammed to iPS cells**	**reprogrammed to iPS cells and EPS cells**	reprogrammed to EPS cells		reprogrammed to naïve hES cells	reprogrammed to naïve hES cells
Maintenance medium	5i/L/A medium on imEFs (Theunissen et al., 2014) or PXGL medium on imEFs (Bredenkamp et al., 2019)	Fibroblast medium	EPSC medium (Yang et al., 2017) on ICR imEFs	EPSC medium (Yang et al., 2017) on CF1 imEFs		PXGL medium on imEFs (Bredenkamp et al., 2019)	PXGL medium on imEFs (Bredenkamp et al., 2019)
Oxygen during maintenance	20%	20%	20%	20%		5%	5%
Platform	AggreWell™ plates	AgreWell™ plates	AggreWell™ plates	AggreWell™ plates		ultra-low attachment multiple-well plate (Corning Coster) + non-adherent, ‘U’-bottomed 96-well (Greiner)	Non-adherent hydrogel microwells (Vrij et al., 2016)
	400 μm Ø	400 μm Ø	400 μm Ø	400 μm Ø			
Human blastoid protocol (Day 0 = seeding on platform)	Day 0	Day 0 Human iBlastoid medium + 10 μM ROCKi	Day −4 to 0 TE induction of EPSC (BMP4 medium)	Day 0 IVF-hEP-hTS (2:1:1) medium + 40 ng/ml FGF2 + TGFβi		Day 0 N2B27 + 1.5 μM MEKi + 1 μM TGFβi + 10 μM ROCKi	Day 0 N2B27+ 10 μM ROCKi
	5i/L/A medium	Day 1 Human iBlastoid medium	Day 0 TE-like cell:EPSC = 5:1∼4:1 EPSC:IVC1 medium = 1.5:1	Day 2 IVF-hEP-hTS (2:1:1) medium + 20 ng/ml FGF2		Day 2 N2B27 + 0.5 μM TGFβi	Day 1 N2B27 + PALLY
						Day 3 N2B27	Day 3 N2B27 + 0.5 μM LPA + 10 μM ROCKi
	HT Day 1 HDM medium	TH Day 1 TDM medium					
	Day 3 TDM medium	Day 6-7 HDM medium					
Oxygen during blastoid formation	20%	5%	20%	5%		5%	5%
Initial cell seeding number	25 cells per microwell	100 cells per microwell	Total 100 cells per microwell (80–83 TSC + 17–20 EPSC)	5–6 cells per microwell		50–200 cells per microwell	3.0 × 104 cells per well (calculated ∼70 per microwell)
Aggregation	12 h	24 h	24 h	24 h		24 h	0–24 h
Cavitation	Day 4	Day 3–4	Day 4	Day 3–4		Day 2	Day 3
	Not present in 85.5% (HT) and 84.3% (TH)						
Specification of tissues	Day 3: GATA6 (HYPO)	Day 1: CDX2 (TE), GATA6 (HYPO) and OCT4 (EPI) cells observed	Day 4: OCT4 (EPI), GATA6 (HYPO)	Day 2–3: GATA3 (TE)		Day 2: GATA3	Day 0-1: DAB2, CDX2, GATA2/3 (TE)
	Day 5: GATA3 (TE)	Day 4: Segregated populations	Day 5–6: GATA2/3, KRT8 (TE)	Day 4: PLAC8, CDX2, KRT8 and KTR18 (TE)		Day 3: KLF17, NANOG, SOX2 (EPI)	Day 2.5: KLF17, OCT4 (EPI)
	Day 7–9: segregation of SOX2 (EPI), GATA3 and GATA6			KLF4, NANOG, OCT4 (EPI)		GATA4, SOX17, OTX2, PDGRFA (HYPO)	Day 3: GATA4, OTX2, SOX17 (HYPO)
				GATA6, PDGFRA and SOX17 (HYPO)			Day 4: NR2F2 (polarTE)
Formation completed	Day 7–9	Day 4 (fully grown on Day 6)	Day 5–6	Day 6		Day 3–4	Day 3–4
Yield of cavitated structures with EPI, TE and HYPO	12.8% (HT) and 9.4% (TH)	5.8%–18% across three donors	1.9%	60%		30%–80%	73%–89%
Embryonic timeline	E6.0-7.0	E5.0–7.0	E6.0	E6.0		E6.0-7.0	E6.0
Characterization	Immunohistochemistry	Immunohistochemistry	Immunohistochemistry	Immunohistochemistry		Immunohistochemistry	Immunohistochemistry
	RT-qPCR	RT-qPCR	RT-qPCR	RT-qPCR		scRNA-seq analysis	RT-qPCR Bulk RNA-seq
	scRNA-seq analysis	scRNA-seq analysis	Bulk RNA-seq	Bulk RNA-seq		Post-implantation progression	scRNA-seq analysis
	Derivation of ES, TS and END cells	hCG ELISA	scRNA-seq analysis	Derivation of ES, TS and END cells			hCG ELISA, pregnancy test
	Post-implantation progression	Derivation of ES, TS and END cells	Derivation of ES and TS cells	Post-implantation progression			Derivation of ES, TS and END cells
	Injection into mouse blastocysts	Post-implantation progression	Post-implantation progression				Post-implantation progression
Markers used	EPI: OCT4, SOX2	EPI: OCT4, SOX2	EPI: OCT4, SOX2	EPI: KLF4, NANOG, OCT4, SOX2		EPI: KLF17, NANOG, SOX2	EPI: KLF17, OCT4, NANOG
	TE: GATA2, GATA3	TE: CDX2, GATA2, KRT8	TE: GATA2/3, KRT8, CK8	TE: CDX2, GATA3, KRT8, KTR18, PLAC8, TFAP2C		TE: GATA3	TE: GATA2/3, CDX2, NR2F2
	HYPO: GATA6, SOX17	HYPO: GAT6, SOX17	HYPO: GATA6	HYPO: FOXA2, GATA6, PDGFRA, SOX17		HYPO: GATA4, SOX17, OTX2, PDGRFA	HYPO: GATA4, S0X17
Post-implantation progression (attached culture)	*In vitro* on μ-Slide 8-well (ibidi) in IVC-1 and IVC-2 medium; start on day 9 of blastoid formation	*In vitro* on optical-grade tissue culture plates in IVC-1 and IVC-2 medium; start on day 9 of blastoid formation	In vitro on 8-well plate (Matrigel treated) in IVC-1 and IVC-2 medium;	In vitro on 96-well ultra-low attachment U-shaped plate (Costar) in mIVC-1 medium; start on day 6 of blastoid formation		In vitro on µ-Slide 8-well (ibidi) coated with Geltrex and cultured in N2B27 medium; start on day 4 of blastoid formation	Implantation assay: In vitro on open-faced endometrial layers
	40%–50% attached and flattened at day 10–11	>90% attached and flattened on day 10	Start on day 6 of blastoid formation	Structures reorganize within 24 h to contain one PODXL+ lumen (pro-amniotic cavity)		Outgrowth from day 2	24–48 h in mIVC-1 medium on top of endometrial 2D culture
	10% formed an pro-amniotic cavity (OTX2+), yolk sac cavity and contained EPI, HYPO and TE lineages on day 12	20%–30% of attached iBlastoids showed EPI-like cell polarization (aPKC) and pro-amniotic cavitation on day 12	GATA6+ (HYPO) cells encircle OCT4+ (EPI) cells on day 8	60% contained SOX2+ (EPI) and KRT18+ and GATA3+ (TE), some also FOXA2 (HYPO)		Outgrowths were largely GATA3+ and CK7+ with some hCGB (TE)	In vitro on suspension 96-well plates for 6 days using monkey blastocyst culture-derived methods
		Outgrowths contained EPI (OCT4+ & NANOG+), HYPO (SOX17+ & GATA6+) and TE (CDX2+, GATA2+ & KRT7+) lineages	Same localization with more cells on day 10	Few GATA4+ (HYPO) and NANOG+ (EPI)		Day 4 after start, OCT4+ (EPI) cluster with a cavity is observed.	
			scRNA-seq comparison with day 8-12 d.p.f. IVC embryos			PODXL+: two lumina (pro-amniotic and yolk sac cavities)	At day 6, EPI cells outnumber TE and HYPO
Limitations	Low yield	Low yield	Low yield	hEPSCs only partly able to specify TE		Variable efficiency	Discontinuous HYPO layer?
	Variable efficiency	No defined HYPO layer	More total cells and fewer cells in the inner cell mass (ICM)	Induced TSCs don’t recapitulate pre-implantation TE, but post-implantation		HYPO cell number varies	Organization of the three lineages during in vitro post-implantation does not reflect in vivo organisation at this stage
	∼80% of cells don’t differentiate or acquire a clear embryonic identity	No signs of gastrulation	∼50% blastoids don’t have correct OCT4+ (EPI) or CK8+ (TE) localization			No evidence of lineage specification potential (no derivation experiment)	
	Low post-implantation progression efficiency	Reported TE more similar to amnion (Zhao et al., 2021)	Large variation in lineage specification				
	Cell loss due to extensive procedure for mEF depletion						

As noted before, human PSCs appear more readily able to contribute to the TE lineage when they are in a naïve or EPSC conditions than mouse PSCs. This might explain why, contrary to murine blastoids, the majority of human blastoids have been formed from a single cell type and higher yields were reported in some of the single cell type approaches (Kagawa et al., 2021; Sozen et al., 2021; Yanagida et al., 2021) compared to the approach of Fan et al. that combines induced TE-like cells with EPSCs (Table 2) (Fan et al., 2021).

### Blastoid Culture Conditions

In addition to 2D PSC cultures in the appropriate pluripotency state, several other conditions must be met to generate blastoids. The PSCs need to be exposed to the right signaling molecules at the right time to direct their differentiation towards the blastocyst lineages and they need the appropriate microenvironment to undergo their morphogenetic changes.

Signaling pathways that are involved in blastocyst formation have been thoroughly studied over the past decades in mouse [reviewed in Rossant and Tam (2009), Frum and Ralston (2015)]. While in mouse, *in vitro* work on the effects of pathway activation can be complemented with *in vivo* work, this is not the case for human due to ethical concerns. Much of what we know about signaling pathways in human development therefore stems from findings in the mouse and/or comparisons between mouse and human ESCs *in vitro*, substantiated with (single cell) omics approaches applied to human (Yan et al., 2013; Blakeley et al., 2015; Petropoulos et al., 2016) and mouse embryos in the recent years (Deng et al., 2014; Boroviak et al., 2015; Nakamura et al., 2016). Based on these studies, we are now able to attribute the activity of several pathways to the development of the blastocyst lineages in both mouse and human. Consequently, small molecules that regulate these pathways are applied in blastoid generation. Here we will discuss all pathway regulators that have reportedly been used so far to generate blastoids and explain their function(s) in blastoid formation (summarized in Tables 3, 4).

**TABLE 3:** Small molecules applied in mouse blastoid protocols. When a protocol is listed in brackets, the molecule is used in a screening or cell culture maintenance medium, but not included in the standard blastoid generation protocol.

Compound name	Pathway	Activator/inhibitor	Promotes	Protocols
(2S)-OMPT	LPA	activator	TE induction	Kime
8Br-cAMP	PKA	activator	PrE induction, self-renewal, swelling	Rivron, Vrij
BMP4	BMP/Smad	activator	cavitation, swelling, TE self-renewal	Kime, Li (, Rivron)
CHIR99201	Wnt	activator	cavitation, pluripotency, PrE induction	Li, Rivron, Sozen, Vrij
(S)-(+)-Dimethindene maleate	M2 muscarinic receptor	inhibitor	pluripotency	(Li,)Sozen
FGF4	FGF	activator	PrE induction, (TE) self-renewal	Li, Rivron, Sozen, Vrij
IL-11	JAK/STAT	activator	decidualization	Rivron, Sozen, Vrij
Insulin	PI3K	activator	proliferation	Li, Rivron, Sozen, Vrij
IWR-endo1	Tankyrase/Wnt	inhibitor	pluripotency	Sozen
LIF	JAK/STAT	activator	cell survival	Kime, Rivron, Sozen, Vrij
Minocycline hydrochloride	PARP	inhibitor	pluripotency	(Li,)Sozen
Retinoic acid	Receptor on the DNA	activator	PrE induction	Vrij
SB431542	TGFβ	inhibitor	TE induction	Kime
TGFβ1	TGFβ	activator	TE-renewal; inhibits swelling	Rivron, Sozen, Vrij
Y-27632	ROCK	inhibitor	cell survival; TSC engulfment	Li, Rivron, Sozen, Vrij

**TABLE 4:** Small molecules applied in human blastoid protocols. When a protocol is listed in brackets, the molecule is used in a screening or cell culture maintenance medium, but not included in the blastoid generation protocol.

Compound name	Pathway	Activator/inhibitor	Promotes	Protocols
b-oestradiol	estrogen receptor	activator	decidualization	Fan, Liu
A83-01	TGFβ (Nodal/Activin/ALK4/5/7)	inhibitor	TE induction	Kagawa, Liu, Sozen, Yanagida
Activin A	TGFβ (Nodal/Activin)	activator	HYPO induction	Yu
BMP4	BMP/Smad	activator	cavitation, TE self-renewal	(Fan,) Liu, Sozen
CHIR99201	Wnt	activator	HYPO induction, pluripotency	Fan, Liu, Sozen, Yu
(S)-(+)-Dimethindene maleate	M2 muscarinic receptor	inhibitor	pluripotency	Fan, Sozen
EGF	Erk/MAPK	activator	TE self-renewal, cavity expansion	Liu, Sozen, Yu
FGF2/bFGF	FGF/Erk	activator	HYPO induction	Sozen, Yu
IM-12	Wnt	activator	pluripotency	Yu
Insulin	PI3K	activator	proliferation	Fan, Liu, Sozen, Yu
IWR-endo1	Tankyrase/Wnt	inhibitor	pluripotency	Fan, Sozen
LIF	JAK/STAT	activator	cell survival	Fan, Kagawa, Sozen, Yu
L-ascorbic acid	antioxidant	-	self-renewal	Liu, Sozen, Yu
Lysophosphatidic Acid (LPA)	Hippo	inhibitor	TE induction	Kagawa
Minocycline hydrochloride	PARP	inhibitor	pluripotency	Fan, Sozen
PD325901	Erk/MAPK	inhibitor	pluripotency	Kagawa, Yanagida
Progesterone	progesterone receptor	activator	decidualization	Fan, Liu
SB431542	TGFβ (Nodal/Activin/ALK4/5/7)	inhibitor	TE self-renewal	Liu, Sozen, Yu
SB590885	BRAF	inhibitor	pluripotency	Yu
VPA	HDAC	inhibitor	TE self-renewal	Liu, Sozen, Yu
WH-4-023	SRC	inhibitor	pluripotency	Yu
Y-27632	ROCK	inhibitor	cell survival	Fan, Kagawa, Liu, Sozen, Yanagida, Yu

### Wnt Pathway Modulators

Wnt signaling is found to play an important role in both mouse and human development, although its role during pre-implantation remains controversial [reviewed in De Jaime-Soguero et al. (2018)]. Regardless of the disputes surrounding Wnt activity during pre-implantation stages, CHIR99201 (CHIR), a GSK3β inhibitor and subsequent canonical Wnt/β-catenin activator, is included in all mouse blastoid protocols except the one from Kime et al. Addition of CHIR significantly increased the yield of mouse blastoid formation (Rivron et al., 2018; Li et al., 2019) and Wnt inhibitors XAV939 and IWR-1-endo significantly inhibited blastoid formation (Rivron et al., 2018; Li et al., 2019). Additionally, it enhanced blastoid cavitation size (Rivron et al., 2018) and PrE induction (Rivron et al., 2018; Vrij et al., 2019).

CHIR is included in several of the human protocols (Fan et al., 2021; Sozen et al., 2021; Yu et al., 2021) and has similar functions as in mouse, namely, to maintain pluripotency of the human PSCs and support HYPO induction. Sozen et al. even found that human 3D cultures did not survive without CHIR (Sozen et al., 2021). However, CHIR is not the only Wnt regulator employed. The naïve pluripotency medium 5i/L/A that is used for blastoid generation by Yu et al. includes the selective GSK3β inhibitor IM-12 (Theunissen et al., 2014). In blastoid protocols based on human EPSCs, EPSC medium rather than naïve medium is used to maintain a pluripotent compartment. EPSC medium also contains CHIR and is paradoxically complemented with a canonical Wnt/β-catenin signaling inhibitor, IWR-endo-1 (Yang Y. et al., 2017; Fan et al., 2021; Sozen et al., 2021), that possibly mediates an effect by further increasing Axin2 levels (Kim et al., 2013).

### ERK/MAPK Pathway Modulators

The ERK/MAPK pathway too is unanimously recognized as a key player. Downregulation of ERK/MAPK signaling was first described in murine ESC cultures to maintain naïve pluripotency alongside Wnt pathway activation (Ying et al., 2008). Through the inhibitor PD0325901, ERK/MAPK signaling is blocked, resulting in sustained pluripotency and self-renewal in both murine and human ESCs (Li et al., 2007; Ying et al., 2008; Nichols et al., 2009). As MEK inhibitors are suppressors of differentiation, they are omitted from mouse blastoid protocols to facilitate lineage specification.

In human however, maintaining ERK/MAPK inhibition in 3D culture supports the specification of the TE lineage and is therefore included in several protocols for human blastoids (Guo et al., 2021; Kagawa et al., 2021; Yanagida et al., 2021). Additionally, the previously mentioned human naïve pluripotency medium 5i/L/A contains both PD0325901 and a BRAF inhibitor, which is also linked to the inhibition of ERK/MAPK signaling (Theunissen et al., 2014).

### TGFβ Pathway Modulators

Then, there are several members of the TGFβ-superfamily that are involved in the regulation of blastoid formation. Most mouse blastoid protocols apply low levels of TGFβ1 to maintain TE self-renewal and modulate swelling of the cavitated structures (Rivron et al., 2018; Sozen et al., 2019; Vrij et al., 2019). Notably, studies in mouse identified TGFβ1 as a co-regulator with FGF4 for TSC self-renewal (Erlebacher et al., 2004). Therefore it is striking that, when murine blastoids are generated from EPSCs alone, they are exposed to Activin/Nodal/ALK4/5/7 inhibitor A83-01 to induce the TE identity in EPSCs (Li et al., 2019). Similarly, Kime et al. apply Activin/Nodal/ALK4/5/7 inhibitor SB431542 in their blastoid protocol (Kime et al., 2019). Regarding the other branch of the TGFβ-superfamily, BMP4, is included in mouse blastoid cultures (Rivron et al., 2018; Kime et al., 2019; Li et al., 2019) to support cavitation, swelling and TE self-renewal (Coucouvanis and Martin, 1999; Hayashi et al., 2010).

Similar to aforementioned murine EPSC-only blastoids and EpiSC-based blastoids, human blastoids are formed in the presence of TGFβ inhibitors. In human naïve PSCs and EPSCs alike, A83-01 in combination with PD0325901 induces a TE identity, while the less potent inhibitor SB431542 is included to support TE self-renewal (Tojo et al., 2005; Okae et al., 2018; Guo et al., 2021). Through the other TGFβ signaling branch, BMP4 contributes to TE self-renewal as well (Xu et al., 2002; Amita et al., 2013; Okae et al., 2018). In one of the human protocols, TE induction before 3D culture is even performed with BMP4 alone (Fan et al., 2021). Whether it can give rise to pre-implantation TE-like cells is unclear, as most studies on BMP4-mediated induction observe trophoblast markers that are expressed by peri- and post-implantation trophoblast cell types (Li and Parast, 2014; Jain et al., 2017; Gao et al., 2019; Fan et al., 2021). Lastly, Activin A, an activator of Activin/Nodal/ALK4/5/7 signaling, is applied in human PSC culture to maintain naïve pluripotency (Theunissen et al., 2014; Bayerl et al., 2021). In combination with Wnt and FGF pathway regulators, Activin A is also used to induce HYPO differentiation (Linneberg-Agerholm et al., 2019; Yu et al., 2021).

### FGF Pathway Modulators

In mice, FGF signaling is most notably involved in directing differentiation of ESCs during pre-implantation. FGF4 specifically is indispensable for mouse ESC differentiation towards the PrE lineage (Kunath et al., 2007; Nichols et al., 2009; Lanner and Rossant, 2010; Yamanaka et al., 2010; Krawchuk et al., 2013). Moreover, FGF4 does not only direct mouse ESCs towards the PrE, but also supports TE self-renewal (Tanaka et al., 1998; Goldin and Papaioannou, 2003). Overall, FGF4, including its chaperone heparin, is an all-round crucial factor for murine blastocyst formation (Allen and Rapraeger 2003; Furue et al., 2008) and therefore it is included in nearly all mouse blastoid models (Rivron et al., 2018; Li et al., 2019; Sozen et al., 2019; Vrij et al., 2019).

In human HYPO development on the other hand, FGF signaling is dispensable, showcasing a marked difference between mouse and human in the regulation of the second lineage bifurcation (Roode et al., 2012). While it might not be essential, FGF signaling through the protein FGF2/bFGF does support HYPO induction, alongside other pathways as mentioned above (Linneberg-Agerholm et al., 2019). Furthermore, it is well-established that FGF2/bFGF supports human ESC self-renewal in the conventional primed pluripotency state (Xu et al., 2005), indicating its importance for maintenance of the post-implantation epiblast.

### JAK/STAT Pathway Modulators

One of the vital factors for mouse ESC proliferation and self-renewal is the JAK/STAT3 pathway activator LIF which is produced by the preimplantation TE and feeds the EPI (Williams et al., 1988). In addition to maintaining pluripotency and EPI identity, LIF also promotes PrE specification in blastocysts (Morgani and Brickman, 2015). A related activator of the JAK/STAT3 pathway, IL-11, complements FGF4 in the maintenance of TE self-renewal in blastoids (Rivron et al., 2018).

Several factors in the JAK/STAT3 pathway are applied in human blastoid protocols as well (Fan et al., 2021; Kagawa et al., 2021; Liu et al., 2021; Sozen et al., 2021; Yu et al., 2021). LIF is widely included to maintain the pluripotent EPI (Fan et al., 2021; Kagawa et al., 2021; Sozen et al., 2021; Yu et al., 2021), even though it appears less crucial for proliferation and self-renewal in human as it is in mouse (Humphrey et al., 2004; Dahéron et al., 2008). Meanwhile EGF is included in several blastoid protocols (Liu et al., 2021; Sozen et al., 2021; Yu et al., 2021) for its support of TE differentiation and self-renewal (Okae et al., 2018).

### ROCK Inhibitor

One universally applied compound in blastoid protocols is the ROCK inhibitor Y-27632. In mouse blastoids, it was found to improve the engulfment of ESC aggregates by TSCs and was subsequently used in all protocols where TSCs are added separately (Rivron et al., 2018). Interestingly, studies in murine blastocysts indicate that ROCK inhibitor prevents cavitation *in vivo* and hampers TE induction through Hippo signaling (Kawagishi et al., 2004; Kono et al., 2014). Whether it similarly obstructs blastoid formation in some way *in vitro* is currently unknown.

For human PSC culture, ROCK inhibitor is a common addition to prevent apoptosis when human PSCs are reseeded as single cells (Watanabe et al., 2007). As such, it is included at least for the first 24 h in every human blastoid protocol to enhance single cell survival before aggregation.

### Hippo Pathway Modulators

The mouse blastoid study by Kime et al. and the human blastoid study by Kagawa et al. show that Hippo signaling inhibition is key for TE specification in blastoids (Kime et al., 2019; Kagawa et al., 2021), similar to mouse (Yu et al., 2016) and human embryos (Gerri et al., 2020a). Kime et al. applied OMPT, an agonist of Hippo inhibitor Lysophosphatidic Acid (LPA) in their phase 2 medium (Kime et al., 2019), after they previously found that LPA promotes the conversion of EpiSCs to naïve PSCs (Kime et al., 2016). In the human blastoid system, Kagawa et al. added LPA and found that this significantly increased the blastoid formation efficiency (Kagawa et al., 2021).

### cAMP Modulation in Mouse Blastoids

In most mouse blastoid models, 8Br-cAMP, a synthetic cyclic AMP analog, is applied in order to support both maintenance of the TE through upregulation of CDX2 expression, as well as to enhance cavitation of the blastoids (Rivron et al., 2018; Sozen et al., 2019; Vrij et al., 2019). This compound has however not been included in any of the human blastoid protocols.

### Modulators Specific for Human Blastoids

Besides signaling pathway modulators with a clear connection to developmental processes, some of the human blastoid protocols include a number of small molecules that have no apparent strong connection to established developmental signaling pathways. Several of these, SRCi, M2-R/H1-Ri, and PARPi, are added to maintain the pluripotent compartment in the blastoids (Theunissen et al., 2014; Yang Y. et al., 2017). The histone deacetylase inhibitor valproic acid (VPA) is included as a component of the previously defined human trophoblast medium (Okae et al., 2018). Notably, the use of either VPA or a similar histone deacetylase inhibitor facilitates the induction of naïve pluripotency *via* resetting of the epigenome (Guo et al., 2017).

### Hormones

Several hormones are also included in some of the models. Insulin has become a standard component of both mouse and human TSC medium compositions and as such is now also present in blastoid protocols of both species (Kubaczka et al., 2014; Okae et al., 2018). Insulin is a well-established activator of the PI3K pathway, through which it promotes cell survival and proliferation (Taniguchi et al., 2006). This support of cell survival and proliferation led to the inclusion of insulin in the media for human PSC cultures, alongside FGF2 and ascorbic acid (Chen et al., 2011). Correspondingly, insulin appears to give naïve human PSCs a growth advantage and thus curbs HYPO differentiation (Anderson et al., 2017). Finally, there are the two sex hormones, β-oestradiol and progesterone, which are known to increase endometrial receptivity in human and mice and upregulate cytokines that support implantation of the murine blastocyst (Norwitz et al., 2001; Basak et al., 2002; Young, 2013). Both hormones have been applied in human blastoid cultures, as components of a medium called “*in vitro* culture 1” (IVC1). This medium is developed for and typically used to permit *in vitro* post-implantation progression of blastocysts rather than for pre-implantation embryo culture (Morris et al., 2012; Bedzhov et al., 2014).

To summarize, there is evidently a myriad of components that have so far been included in blastoid protocols, especially in the human ones. This can be attributed to the fact that most of the currently published human protocols use mixes of pre-existing media to induce the blastocyst lineages in 3D cultures. Notably, the protocols developed by Yanagida et al. and Kagawa et al. only include a handful of pathway regulators and are therefore surprisingly minimalistic compared to the others. This begs the question which of the compounds applied so far are truly indispensable for human blastoid generation, particularly when using PSCs maintained in the naïve state.

### Culture Platforms

Regulating the mechanical environment of stem cells to direct assembly, trigger differentiation and guide development has recently gained traction, since an increasing number of studies show a relation between physical properties of the microenvironment and stem cell behavior (Guilak et al., 2009; Han et al., 2014). For a review on the potential of *in vitro* models to study mechanical and geometrical cues in early mammalian embryogenesis, refer to (Vianello and Lutolf, 2019).

In the case of blastoids, the culture platforms need to facilitate several requirements. Firstly, after initial seeding, cells need to be in each other’s proximity to aggregate. Secondly, the cells/aggregates require the efficient exchange of nutrients, signaling molecules and growth factors to support their growth, differentiation and proliferation. Thirdly, it is important to take into account that mechanical cues also directly affect the signaling pathways involved in development (e.g., Hippo signaling) (Barzegari et al., 2020). As blastoids are a pre-implantation model, adherence to the culture platform should be prevented. Usually, this is established either through the use of a suitable, non-adherent material [e.g., agarose or polyethylene glycol (PEG) for hydrogel microwells], by pre-coating culture plates with an anti-adherence solution [e.g., polyethylene-oxide polypropylene-oxide (hydrophilic-hydrophobic)] block copolymers for Aggrewells or by using pre-treated plates (ultra-low attachment plates). Finally, through the use of microwells arrays, large quantities of blastoids can be produced for one experiment, facilitating high-throughput analysis.

For murine blastoids, either in-house produced agarose hydrogel microwells or commercial Aggrewell microwells have been used (Table 1). Both set-ups contain a large quantity of microwells (400–1200 per well of a well-plate) (Vrij et al., 2016). Advantages of agarose hydrogel microwells over hard-plastic Aggrewell microwells may be the aid in diffusion of nutrients and waste products throughout the gel and the circular geometry that is less obstructive compared to the inverted pyramidal-shape of Aggrewell microwells. Moreover, agarose hydrogel microwells are amenable to *in situ* bright-field and epi-fluorescence imaging and downstream analysis and therefore blastoids do not require transfer to other platforms prior to analysis. The disadvantage of agarose-hydrogel microwells however is that diffusion of soluble components into the gel hampers abrupt and complete switches in medium conditions. Additionally, transfer of blastoids is still required for high resolution (i.e., confocal-based) imaging.

Similar to mouse, most human blastoid protocols are developed using Aggrewell microwells. However, Kagawa et al. used the agarose hydrogel microwells and Yanagida et al. reported using ultra-low attachment multiwell plates and non-adherent, “U”-bottomed 96-wells plates (Table 2). Yanagida et al.’s method of manually transferring structures to new media conditions however is very laborious, time-consuming, likely leading to the loss of (developing) blastoids and not amenable to high-throughput screenings.

Although Aggrewell microwells are widely used and support the culture of more than 1000 structures per well, they too come with several disadvantages. While it may seem arbitrary, a technical issue due to their pyramidal shape is the difficulty of changing media without disturbing the forming structures. As such, switching media for sequential inductions of blastocyst lineages is practically hindered. In contrast, agarose hydrogel microwells are far less sensitive for flow disturbances due to their cylindrical geometry. Another disadvantage of Aggrewell microwells is that the inverted pyramidal shape significantly hinders the *in situ* imaging quality and thus image-based readouts of the structures. As a result, blastoids need to be manually transferred to different platforms for imaging and further analysis, which poses the same issues as listed above for the method employed by Yanagida et al. All in all, among the currently reported microwell platforms for blastoid generation, a platform in which blastoids can be imaged directly with high quality is still lacking.

### Recapitulation of Development

The formation of blastoids consists of roughly the same steps in mouse and human protocols: aggregation, cavitation and maturation. First, upon seeding, the PSCs need to aggregate into spherical structures. This invariably occurs in the first 24 h with both mouse and human cells. Generally, media still mainly contain factors to support maintenance of pluripotency at this stage. Once the PSCs have clumped together, the medium composition is often changed to induce lineage specification and self-renewal of these lineages. For methods that require TSCs separately, TSCs are added after the first 24 h. Subsequently, the TSCs engulf the aggregated PSCs. From this stage onward, the forming blastoid structures self-organize and undergo cavitation, followed by a maturation process in which the blastoid increases in size, forms distinct EPI and PrE cells and occasionally organize themselves by sorting out a continuous epithelial layer of PrE cells overlying the EPI within the blastoid. Lineage specification takes place in parallel with these morphological changes. Thus, blastoids undergo embryonic events reminiscent of the pre-implantation embryo (Takaoka and Hamada, 2012).

*In vivo,* mouse and human development occur at a different pace. While the mouse embryo grows from a zygote to a blastocyst in approximately 3.5 days and implants around day 4.5–5, the human embryo reaches the blastocyst stage around day 5 and implants between day 7 and 8 (Molè et al., 2020). Interestingly, this difference in developmental pace between species can to some extent also be observed *in vitro*. Mouse blastoids generated with naïve ESCs have been reported to cavitate as early as 48 h after adding TSCs to ESC aggregates, while protocols using mouse EPSCs report cavitation around 72–96 h post TSC seeding. When using EpiSCs, cavitation may even occur as late as after 120 h, however this protocol is difficult to compare with the others, since the 3D structures arise from a converting 2D culture, rather than a 3D setup (Table 1). Similar to mouse EPSC-based blastoids, most human blastoids form a cavity between 72 and 96 h (Table 2). The blastoids from Kagawa et al. and Yanagida et al. based on naïve PSCs deviate from this timing. These blastoids were observed to have a cavity as early as at the 48-h time point (Yanagida et al.) or at the 60-h time point (Kagawa et al.).

When blastoids contain all three lineages and their morphology resembles the blastocyst, they are considered fully formed and their generation is complete. At what time point this stage is reached varies hugely between models. Mouse blastoids are generally fully formed at 96 h, except for the Li et al. EPSC-only blastoids, which are fully formed at 120–144 h, and the EpiSC-based blastoids from Kime et al., which may take up to 168 h. Completion of human blastoid generation on the other hand varies between 96 and 216 h. While naïve PSC-based blastoids by Yanagida et al. and Kagawa et al. are fully formed in 96–120 h (Kagawa et al., 2021; Yanagida et al., 2021), similar to the timeline for human morula to blastocyst development (Niakan et al., 2012; Rossant and Tam, 2017), Yu et al. report that their protocol, also based on naïve PSCs, takes 7–9 days (168–216 h) (Yu et al., 2021). The iBlastoids from Liu et al. and both EPSC-based blastoid models require approximately 144 h (Fan et al., 2021; Liu et al., 2021; Sozen et al., 2021). The difference in timing between mouse and human likely reflects the different rates of development also observed between the blastocysts of these species*.* The variation observed between models of the same species on the other hand may reflect the efficiency of PSC commitment to the blastocyst lineages and the efficiency of the subsequent interactions between lineages.

It is precisely the proper commitment of PSCs to the blastocyst lineages that is one of the general challenges in both mouse and human blastoid models. In mouse models, the initial naïve ESC-based blastoids often lack a well-defined PrE compartment (Rivron et al., 2018). Subsequent studies led to a modified protocol that specifically induces PrE formation in blastoids (Vrij et al., 2019). Notably, blastoids based on EPSCs form PrE more readily, which Sozen et al. linked to the enhanced pluripotency state of EPSCs compared to mouse ESCs (Sozen et al., 2019). Despite this enhanced potential, EPSC-only blastoids from Li et al. contain mislocalized EPI/PrE- or TE-like cells as well as significant cell populations that might consist of either uncommitted PSCs remaining in the EPSC state or intermediates between lineages (Li et al., 2019).

The presence of cell types *in vitro* that are not present in the natural blastocyst is usually ascribed to incomplete induction. These off-target cell types can either be entirely uncommitted to any lineage, intermediates between EPI, PrE/HYPO and/or TE, or they correspond to a later stage of development. For many of the current human blastoid models, either one or multiple types of off-target cells have been described. Further inspection of lineage markers in the iBlastoids led to the conclusion that the majority of the cells in these structures are not conclusively committed to a specific lineage, but express markers from at least two different lineages (Liu et al., 2021; Yanagida et al., 2021). Similar overlap in marker gene expression was observed by Fan et al. in their EPSC-based blastoids (Fan et al., 2021). The naïve cell-based blastoids from Yu et al. also contain a large fraction of off-target cells, but these seemingly remain in a naïve-like, uncommitted state (Yanagida et al., 2021; Yu et al., 2021). These uncommitted cells most resemble EPI and far outnumber the TE-like cells, while in the blastocyst TE is the most abundant of the three lineages (Yanagida et al., 2021). In contrast, Yanagida et al. showed that the majority of cells in their blastoids commit to the TE lineage. This is confirmed by the mutually exclusive expression of GATA2 in their TE compartment and expression of OCT4 and naïve marker KLF17 in the ICM compartment. In the same line, Kagawa et al. show that between 50 and 80% of the cells in their blastoids can be attributed to the TE lineage based on immunofluorescence staining. Additionally, they report that less than 3% of cells were transcriptionally similar to post-implantation tissues (amnion and extra-embryonic mesoderm), when comparing the single cell transcriptomes of blastoid cells to blastocysts, *in vitro* outgrowths of blastocysts and gastrulation-stage embryos (Petropoulos et al., 2016; Zhou et al., 2019; Tyser et al., 2020). All other cells matched pre-implantation blastocyst lineages.

While blastoids from Yu et al. contain cells stuck in a developmental stage preceding the blastocyst stage, the iBlastoids appear to contain cells that correspond to the post-implantation stage. When comparing their single cell transcriptomic data with post-implantation blastocysts, the cell population within iBlastoids that was identified as TE is found to more closely resemble amnion-like cells, rather than TE (Zhao et al., 2021). This could suggest that the cells used to make iBlastoids are not completely reprogrammed towards a state of pluripotency similar to naïve PSCs and therefore less able to give rise to the TE compartment (Zheng et al., 2019; Cinkornpumin et al., 2020; Guo et al., 2021). Sozen et al. found that their human EPSC-based blastoids too have only partially specified TE, as the TE compartment could not form a cohesive epithelium. Moreover, some of these EPSC -based blastoids contain multiple cavities (Sozen et al., 2021). Besides the specification of the TE, current human blastoid models also struggle with formation of the HYPO compartment. As was the case for the first mouse blastoids (Rivron et al., 2018), the human models often appear to lack the proper localization and/or HYPO cell numbers (Fan et al., 2021; Liu et al., 2021; Sozen et al., 2021; Yanagida et al., 2021; Yu et al., 2021).

## Comparison of Blastoids With Blastocysts

In order to prove the functionality of blastoids as models of blastocysts, two experiments are generally conducted. First, following the identification and correct spatial allocation of the EPI, PrE/HYPO and TE lineages (e.g., through single cell RNAseq and immunohistochemistry), cell lines are derived from these cell types as a functional assay to verify their propagation potential *in vitro* and their downstream differentiation potential. The second experiment is to test the capacity of complete blastoids to undergo post-implantation progression.

### Derivation of Stem Cell Lines

Originally developed for derivation from natural mouse blastocysts, the general approach to obtain stem cell lines from blastoids is to seed the blastoids on a culture plate with either ESC, TSC or PrE/hypoblast-like cell culture medium (Evans and Kaufman, 1981; Martin, 1981; Tanaka et al., 1998; Ying et al., 2008; Niakan et al., 2013; Okae et al., 2018; Linneberg-Agerholm et al., 2019) that selectively favors the desired cell type over several passages. The mouse naïve PSC-based blastoids of Rivron et al., the mouse EPSC-only blastoids of Li et al. and mouse EpiSC-based blastoids of Kime et al. all confirmed the presence of EPI and TE lineages in their blastoids with this approach (Rivron et al., 2018; Kime et al., 2019; Li et al., 2019). Additionally, blastocyst chimera assays confirmed the contribution of these cell lines to the expected compartments when grown out *in utero.* Moreover, besides the chimeric contribution of EPI and TE lineages, the PrE line derived from mouse EPSC-only blastoids contributed to the yolk sac.

As for the human blastoid models, derivation experiments were performed on the naïve PSC-based blastoids of Kagawa et al. and Yu et al., the EPSC-based blastoids of Fan et al. and the iBlastoids of Liu et al., though only Yu et al. included a culture selecting for HYPO-like cells (Linneberg-Agerholm et al., 2019; Yu et al., 2021). Remarkably, despite the low number of TE-like cells, Yu et al. was able to derive TSC cells from single plated blastoids, as well as naïve PSCs and naïve endoderm cells. The identity of these cell lines was confirmed with immunostaining, differentiation assays and injection of the derived cells into mouse blastocysts. Kagawa et al. were able to derive naïve PSCs and TSCs from their blastoid model. These derived TSCs differentiate towards syncytiotrophoblast and extravillous trophoblast cell fates in 3–6 days, as was confirmed through immunostaining and RT-qPCR of several markers. Strikingly, blastoid-derived naïve PSCs could be used to generate a second generation of blastoids. Also Liu et al. derived TSCs and naïve PSCs from their iBlastoids. The naïve PSCs were confirmed to be pluripotent through a tri-lineage differentiation assay and the TSCs were differentiated towards TE derivatives, with immunostainings supporting the presence of marker genes. Finally, Fan et al. derived PSCs and TSCs from their blastoids, which they confirmed with immunostainings of several lineage markers.

Though these derivations say little about the developmental potential of the blastoids as complete structures, they do indicate that blastoids contain PSCs or cells with similar potential capable of differentiating towards post-implantation cell types.

### Post-Implantation Development *In Vitro*

Culture media that permit the *in vitro* post-implantation progression of blastocysts have in recent years contributed to embryo phenotyping and mechanistic understanding, particularly for human since this stage is non-accessible *in vivo* (Bedzhov et al., 2014; Deglincerti et al., 2016; Shahbazi et al., 2016; Xiang et al., 2020).

*In vitro*, blastocysts are transferred to a plastic or glass substrate and cultured in optimized *in vitro* culture (IVC) medium containing for the first 2 days serum, to promote growth and attachment of the TE cells, which is later substituted by knockout serum (Bedzhov et al., 2014). With this method, post-implantation morphological changes in the embryo, in particular the EPI transformation into the egg cylinder with concomitant visceral endoderm patterning, can easily be tracked for up to 5 days of development. Likewise, blastoids can be exposed to similar culture conditions to assess their potential for early post-implantation development. Naïve mouse ESC-based blastoids reportedly only continue development rarely in the *in vitro* system. The PrE-induced blastoids on the other hand manage to occasionally grow out into post-implantation epiblast-like structures encasing pro-amniotic-like cavities- and surrounded by a visceral endoderm-like epithelium (Vrij et al., 2019). Vrij et al. suggested this developmental potential is dependent on the abundance of PrE-like cells in the blastoids prior to post-implantation culture (Vrij et al., 2019). This is supported by the *in vitro* post-implantation assays performed with EPSC-based blastoids, which form PrE more robustly. In line with the development of blastocysts and post-implantation embryo models (Bedzhov et al., 2014), both EPSC-based mouse blastoid models could form egg cylinder-like structures (Li et al., 2019; Sozen et al., 2019). These structures contained cells similar to EPI, and post-implantation PrE and TE derivatives, visceral endoderm and extraembryonic ectoderm, respectively. Moreover, Li et al. observed that these structures recapitulated post-implantation polarization events on the molecular level (Li et al., 2019). Importantly, however, current 2D *in vitro* culture methods for post-implantation morphogenesis of blastocysts and blastoids do not readily permit the formation of mural trophectoderm, associated parietal endoderm and the ectoplacental cone [current gaps in embryo model development are reviewed in Shankar et al. (2021)].

The golden standard for mouse models however, is the injection of blastoids into the uteri of pseudo-pregnant mice to assess their capacity for full placentation and development beyond implantation. Rivron et al. reported the ability of naïve ESC-based blastoids to induce decidualization *in utero* (between 10 and 20% of injected blastoids implanted)*,* but structures failed to develop further, similar to the *in vitro* findings. The EPSC-only blastoids of Li et al. resembled naïve ESC-based blastoids in that they could implant in the uterine wall and trigger decidualization (with ∼7% efficiency) but failed to continue development in this more stringent assay (Li et al., 2019). Sozen et al. reported similar findings for their EPSC-based blastoids. Upon closer inspection, they found that although the blastoids physically attached to the maternal tissue, they did not develop a Reichert’s membrane (Sozen et al., 2021). This membrane was found to play an important role in post-implantation morphogenesis (Ueda et al., 2020), therefore the lack of this structure in the post-implantation progression of blastoids may prevent further development.

Although it is possible to perform *in vivo* post-implantation assays for mouse blastoids, this is obviously unacceptable for the human equivalent. Therefore, human blastoids were only tested for their post-implantation developmental potential *in vitro*. Most groups use roughly the same attached culture method as described above for mouse blastoids and blastocysts (Bedzhov et al., 2014), adapted to support human blastocyst development *in vitro* (Deglincerti et al., 2016). Notably, all the groups that applied this method report a flattening of the blastoid structures upon attachment as is also observed in *in vitro* post-implantation blastocyst culture (Deglincerti et al., 2016; Shahbazi et al., 2016). Moreover, all confirm the presence of markers of at least two of the three blastocyst lineages and provide evidence of the onset of amniotic cavity formation. Additionally, Yu et al. and Yanagida et al. report indications of the onset of yolk sac formation. However, the success rates for the formation of post-implantation structures are quite low. Yu et al. obtained post-implantation-like structures from 10% of the blastoids, while 20–30% of the iBlastoids plated for attached culture formed these structures. Sozen et al. confirmed that ∼60% of their attached structures had clear EPI and TE compartments, but this rate is lower for structures that also contain a HYPO marker. What fraction of these post-implantation structures contains a pro-amniotic-like cavity is not quite clear. Meanwhile Fan et al. and Yanagida et al. reported post-implantation-like structures as well but did not include the formation efficiency.

Kawaga et al. had a slightly different approach for mimicking post-implantation progression. They applied a method similar to the common one described above, but theirs was adapted from a protocol designed for cynomulgus monkey embryos, rather than mouse (Ma et al., 2019). Using this attached culture method, they reported the differentiation and expansion of all three blastocyst lineages and the formation of an amniotic cavity in some attached blastoids. Their post-implantation culture could be maintained for 6 days, to a day 13 equivalent stage, however these cultures did not recapitulate the spatial organization of this developmental stage *in vivo*.

Taken together, the results of the assays discussed above indicate that both the current mouse and human blastoid models are capable of mimicking some of the morphogenetic events typical for the post-implantation blastocyst *in vitro*, albeit at a low efficiency. However, the more stringent *in vivo* experiments with mouse blastoids and the failure of human blastoids to comprehensively recapitulate spatial tissue organization of post-implantation development, underline that the developmental potential of current blastoid systems and/or post-implantation culture methods is still limited for both species. More sophisticated bioengineered 3D culture methods may be needed to overcome this barrier.

### Modelling Processes of Blastocyst Development

Besides investigations of the potential of blastoid models to continue development to a post-implantation stage, some groups reported additional findings to showcase the capacity of their blastoid models to recapitulate essential processes during pre-implantation development.

In mouse blastoids, Rivron et al. found that the EPI maintains the self-renewal and epithelial identity in the adjacent polar TE (Rivron et al., 2018). This is in line with findings from Gardner et al. that trophoblast cells in contact with the ICM don’t terminally differentiate, but instead contribute to the proliferating polar TE-derived ectoplacental cone (Gardner and Johnson, 1972; Gardner et al., 1973).

Furthermore, Li et al. demonstrated that their EPSC-only blastoids accumulated ZO1 and E-Cadherin at cell-cell junctions during the aggregation of the EPSCs which resembles compaction in the 8-cell stage. They also provide evidence of apical enrichment of PARD6 expression during blastoid formation (Li et al., 2019), which is an indication of polarization (Chazaud and Yamanaka, 2016). Additionally, they show nuclear YAP expression in the outer cells and cytoplasmic YAP expression in the inner cells in about 60% of their EPSC-only blastoids on day 5 (Li et al., 2019). This intracellular localization pattern of YAP, part of the Hippo signaling, is proposed to play a role in TE specification in human and mouse during blastocyst formation (Gerri et al., 2020a).

Similar evidence of processes preceding blastocyst formation *in vivo,* such as compaction and polarization, was found in human blastoids as well. Sozen et al. observed the baso-lateral expression of E-cadherin and apical enrichment of F-actin and PARD6 (Sozen et al., 2021). In previous studies, the PLC-PKC pathway was suggested to play a role in cell polarization and TE specification in human embryos (Zhu et al., 2020). Using their blastoid system, Sozen et al. could observe that both PLC-specific inhibition and depletion reduced the expression of GATA3, one of the early TE markers, in outer cells of aggregates (Sozen et al., 2021). Simultaneously, the apical expression of PARD6, which marks polarization, was reduced too. Natural human embryos have a comparable response to *in vitro* PLC inhibition and depletion treatments (Zhu et al., 2020), supporting the capacity of the blastoid system to recapitulate pre-implantation processes.

Furthermore, Sozen et al. investigated the effect of WNT3A. In mouse blastoids, WNT3A supports cavitation and increased blastoid yield (Rivron et al., 2018). However, addition of WNT3A does not improve cavitation in human blastoid cultures. This may indicate a different role for WNT3A in human development (Sozen et al., 2021).

In line with the study of the PLC-PKC pathway by Sozen et al., Yu et al. more extensively investigated the presence of tight junctions in their blastoids and found that, similar to murine blastocysts (Eckert et al., 2004a; Eckert et al., 2004b), their blastoids required the activity of specific PKC for cavitation (Yu et al., 2021).

Kagawa et al. further complimented these findings by their study of Hippo signaling in their blastoids. They found that atypical PKC inhibition strongly reduced nuclear accumulation of YAP1. As aforementioned, as a downstream effector of Hippo signaling, YAP1 is widely established to be involved TE specification in both mouse and human *in vivo* (Gerri et al., 2020a). Kagawa et al. confirmed the same is true in their blastoids, as aPKC inhibition and reduced YAP1 nuclear accumulation also correlated with reduced number of GATA3-positive cells and overall failure of human PSC aggregates to form blastoids. In addition, they show that YAP1 overexpression accelerated cavitation of their blastoids (Kagawa et al., 2021). They further elucidated on the process of cavitation by demonstrating that, similar to the mouse blastocyst (Dumortier et al., 2019), the cavity of their blastoids forms through the merging of multiple fluid-filled cavities (Kagawa et al., 2021).

Beyond the processes of blastocyst formation prior to implantation, blastoids can be used to model the earliest maternal-embryo interaction. In fact, Kagawa et al. present an *in vitro* model of embryo adhesion, the first step in implantation, by combining their blastoid model with so-called open-faced endometrial layers (OFELs) that mimic the endometrium. Using this set-up, Kagawa et al. demonstrate that their blastoids are capable of attaching to receptive endometrium cells and repelling it. Moreover, they show blastoids specifically attach to OFELs with their polar TE region. This is the region of the TE that directly borders on the EPI cluster. Kagawa et al. generated several types of trophospheres, which are blastoids without an EPI compartment, and none of these trophospheres were unable to attach to receptive OFELs, neither did post-implantation stage TSCs and human PSC aggregates. Thus Kagawa et al. provide evidence that the TE receives cues from the EPI that enable it to interact with the endometrium. *In silico* ligand-receptor pair analysis performed by Kagawa et al. using single-cell transcriptomics data yielded a list of potential molecular interactions between TE and endometrium epithelium, which may provide clues for future studies on implantation.

All in all, both mouse and human blastoids, despite the abundance of off-target cell types detected, especially in some of the current human models, already show great potential for investigating pre-implantation processes, such as polarization and cavitation. Notably, Kagawa et al. demonstrated that human blastoids can be used to model implantation *in vitro* when combined with maternal endometrium epithelium cell types. Although it remains unknown whether blastoids fully mimic pre-implantation development while their lineage specification remains (partially) incomplete, current results indicate that some processes underlying blastocyst formation *in vivo* may be faithfully recapitulated *in vitro.*

## Conclusion and Prospects

There can be little doubt that blastoids are a valuable addition to the collection of stem cell-based models. Compared to other stem cell-based embryo-like structures, including gastruloids, 2D micropatterned stem cells, post-implantation amniotic sac embryoids and embryonic-extraembryonic fusion embryoids [reviewed in Fu et al. (2021), Shankar et al. (2021)], blastoids have the unique potential to recapitulate the pre-implantation embryo and are therefore the singular model for studying this early stage of development *in vitro.*

The development of mouse blastoids several years ago has paved the way for the recent development of the human blastoid model. Although there are clear differences between mouse and human in terms of signaling pathway regulation, they share similar core signaling pathways that are tightly regulated during early embryogenesis. This knowledge has guided studies towards medium compositions that are now used to induce the three founding blastocyst lineages in the human blastoid. Moreover, microwell culture platforms first applied to mouse models, as well as several lineage markers originally identified in mice have been adopted for the human system as well. Improvements are still required for blastoids to more convincingly mimic peri- and post-implantation development, particularly regarding the extra-embryonic mesoderm, HYPO and TE compartments. Nevertheless, the first results are promising, as they show that the current models undergo morphological changes and upon attachment partly mimic the architecture of post-implantation embryonic and several extra-embryonic structures *in vitro*. More importantly, pre-implantation development is at least partially recapitulated and blastoids can be used to study cell-cell interactions typical for the blastocyst stage as well as molecular pathways involved in lineage specification.

The possibility of mimicking the pre-implantation embryo has huge implications for a plethora of studies, such as aneuploidy studies, toxicity screens, development of improved IVF compounds and studies on peri-implantation cell differentiation. Combined with more sophisticated, bioengineered *in vitro* models of the uterine wall, human blastoids may become an attractive tool for studying the process of implantation in humans. New insights in this area will indubitably deepen our understanding of key requirements for successful implantation and subsequently aid the development of new contraceptives and therapeutic routes towards treating implantation failure in the future.

The introduction of the human blastoids to the arena of stem cell-based embryo models does certainly not make the mouse models redundant, however. On the contrary, increasingly meaningful comparisons can be made as these models are further developed to more faithfully recapitulate early embryogenesis. Comparisons between mice and human embryogenesis are relevant from an evolutionary perspective, but may also serve the field of medicine, particularly when considering implantation-related complications, as mentioned above. The more extensively studied mouse model may provide more clues for specific pathways and mechanisms to probe in the human system, saving both time and human material. Moreover, while the first reports of human blastoids have sparked debates on the ethical restrictions on research performed with human embryo (-like) structures (Pereira Daoud et al., 2020; Clark et al., 2021), the mouse embryo models may remain the favored models for more ethically challenging experiments, e.g., involving gene-editing techniques. Additionally, mouse models may be able to help chart post-implantation to a stage that human models will not be allowed to reach for ethical and legal reasons, such as *in vitro* and *in utero* advanced organogenesis or organismal development. In conclusion, these two model systems will continue to develop alongside each other and together have broad future application, both for fundamental and clinical research.
